# Randomized controlled trial of the “WISER” intervention to reduce healthcare worker burnout

**DOI:** 10.1038/s41372-021-01100-y

**Published:** 2021-08-09

**Authors:** Jochen Profit, Kathryn C. Adair, Xin Cui, Briana Mitchell, Debra Brandon, Daniel S. Tawfik, Joseph Rigdon, Jeffrey B. Gould, Henry C. Lee, Wendy L. Timpson, Martin J. McCaffrey, Alexis S. Davis, Mohan Pammi, Melissa Matthews, Ann R. Stark, Lu-Ann Papile, Eric Thomas, Michael Cotten, Amir Khan, J. Bryan Sexton

**Affiliations:** 1 Division of Neonatal and Developmental Medicine, Department of Pediatrics, Stanford University School of Medicine and Lucile Packard Children’s Hospital, Palo Alto, CA USA; 2 California Perinatal Quality Care Collaborative, Palo Alto, CA USA; 3 Department of Psychiatry, Duke University School of Medicine, Duke University Health System, Durham, NC USA; 4 Duke Center for Healthcare Safety and Quality, Duke University Health System, Durham, NC USA; 5 Duke University School of Nursing, Durham, USA; 6 Department of Pediatrics, Duke University School of Medicine, Durham, USA; 7 Division of Pediatric Critical Care Medicine, Department of Pediatrics, Stanford University School of Medicine and Lucile Packard Children’s Hospital, Palo Alto, CA USA; 8 Department of Biostatistics and Data Science, Wake Forest School of Medicine, Winston-Salem, NC USA; 9 Department of Neonatology, Beth Israel Deaconess Medical Center, Boston, MA USA; 10 Division of Neonatal-Perinatal Medicine, University of North Carolina Chapel Hill School of Medicine and University of North Carolina Children’s Hospital, Chapel Hill, NC USA; 11 Section of Neonatology, Baylor College of Medicine and Texas Children’s Hospital, Houston, TX USA; 12 Department of Pediatrics-Neonatology, The University of Texas Health Science Center and Children’s Memorial Hermann Hospital, Houston, TX USA; 13 Department of Pediatrics, Division of Newborn Medicine, Harvard Medical School, Boston, MA USA; 14 Division of Neonatology, Department of Pediatrics, The University of New Mexico School of Medicine, Albuquerque, NM USA; 15 Department of Internal Medicine, The University of Texas Health Science Center and Memorial Hermann Medical Center, Houston, TX USA; 16 Division of Pediatrics-Neonatology, Duke University School of Medicine and Duke University Hospital, Durham, NC USA

**Keywords:** Outcomes research, Quality of life

## Abstract

**Objective:**

Test web-based implementation for the science of enhancing resilience (WISER) intervention efficacy in reducing healthcare worker (HCW) burnout.

**Design:**

RCT using two cohorts of HCWs of four NICUs each, to improve HCW well-being (primary outcome: burnout). Cohort 1 received WISER while Cohort 2 acted as a waitlist control.

**Results:**

Cohorts were similar, mostly female (83%) and nurses (62%). In Cohorts 1 and 2 respectively, 182 and 299 initiated WISER, 100 and 176 completed 1-month follow-up, and 78 and 146 completed 6-month follow-up. Relative to control, WISER decreased burnout (−5.27 (95% CI: −10.44, −0.10), *p* = 0.046). Combined adjusted cohort results at 1-month showed that the percentage of HCWs reporting concerning outcomes was significantly decreased for burnout (−6.3% (95%CI: −11.6%, −1.0%); *p* = 0.008), and secondary outcomes depression (−5.2% (95%CI: −10.8, −0.4); *p* = 0.022) and work-life integration (−11.8% (95%CI: −17.9, −6.1); *p* < 0.001). Improvements endured at 6 months.

**Conclusion:**

WISER appears to durably improve HCW well-being.

**Clinical Trials Number:**

NCT02603133; https://clinicaltrials.gov/ct2/show/NCT02603133

## Introduction

Burnout is characterized as a state of depletion, detachment, and cynicism resulting from prolonged high levels of stress [1]. Health care workers (HCWs) in general, especially critical care workers, are at risk for burnout [2, 3], fueled by changes in technology and guidelines, endeavors for high-quality care, and emotional challenges of dealing with critically ill patients and their families [4–6]. Emotional exhaustion alone, one of three domains of burnout, affects 25–50% of neonatal intensive care unit (NICU) HCWs [1, 5], with up to half of nurses and physicians across specialties meeting criteria for severe burnout [7–9]. Burnout among HCWs has been linked to adverse patient events, including increased rates of infections [10, 11], and self-reported errors [10, 12]. Furthermore, burnout may lead clinicians to drop out of the workforce, increasing costly turnover [13, 14] and staffing shortages [15].

Feasible interventions to alleviate burnout are few, and none have been tested and reported in the NICU setting [9]. Since 2011, we have developed and refined an interactive, low-burden program (web-based implementation for the science of enhancing resilience (WISER)) to target enduring reductions in burnout. This stepwise program uses updated versions of evidence-based interventions drawn from positive psychology that have been effective in improving well-being and reducing depression symptoms, delivered via mobile platform [16–19]. WISER components are sequenced purposefully to maximize participant engagement and learning. Components gradually encourage participants to first notice and savior positive emotions and then to act to elicit them. Reminders promote mastery through practice.

Use of and access to well-being interventions must be easy and engaging in order to be utilized by busy HCWs. Our objective was to test the efficacy of WISER in improving NICU HCW burnout (primary outcome), depression, work-life integration, and happiness (secondary outcomes):

Hypothesis 1: Efficacy of WISER: the intervention will improve HCW burnout (the primary outcome is emotional exhaustion), depression, work-life integration, and happiness (secondary outcomes) in cohort 1 compared with waitlist control in cohort 2 by the 1-month post-intervention primary endpoint.

Hypothesis 2: WISER will be effective at 1-month post-intervention.

Hypothesis 3: Effect of WISER will endure at 6-months post-intervention.

Hypothesis 4: The condensed cohort 2 intervention will not be less effective than the full intervention for cohort 1.

## Methods

### Design

**Fig. 1:**
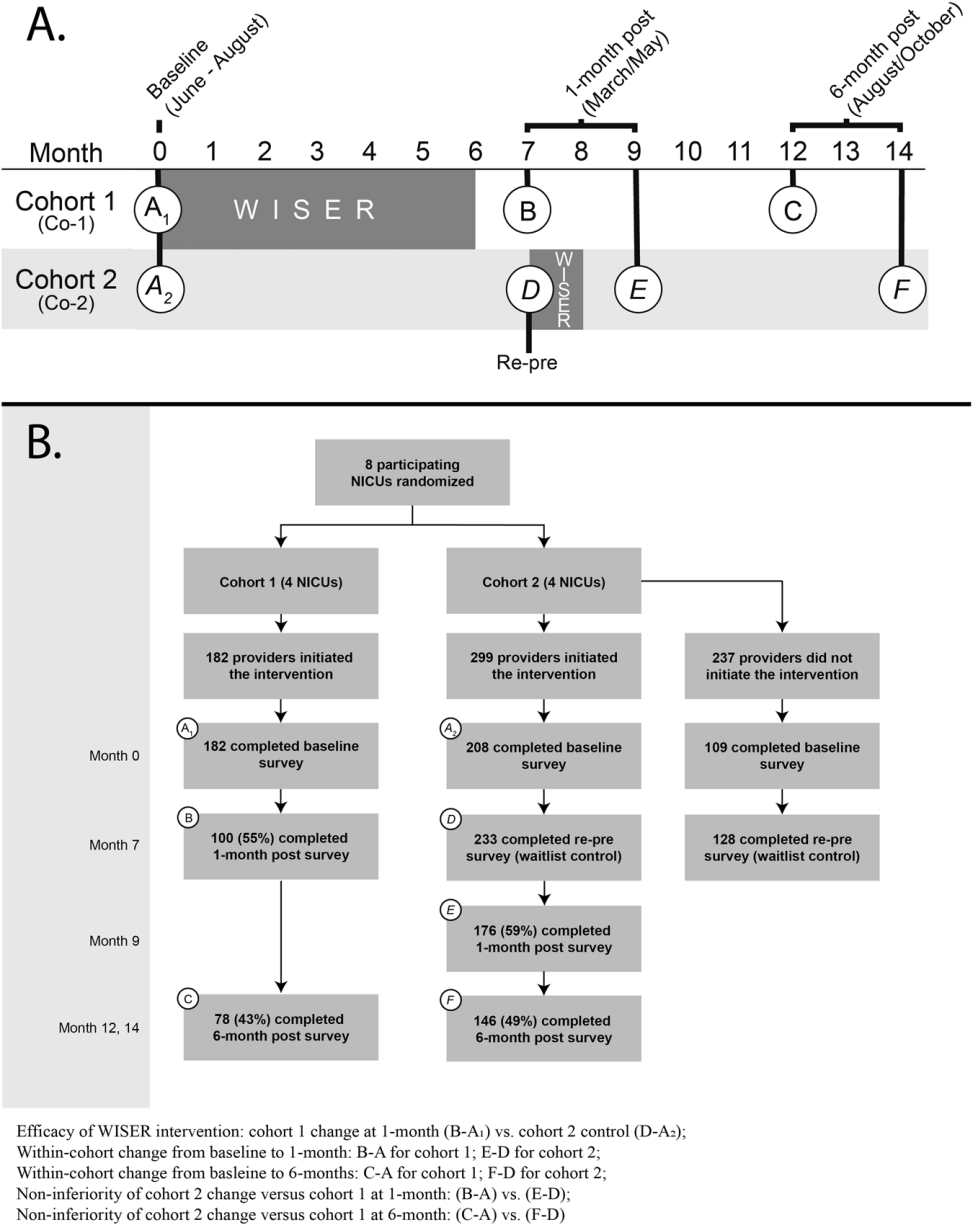
**A** Schematic of WISER study design. Active WISER period shaded gray. Seven assessment points presented in circles: two baseline assessment (A_1_ and A_2_), two 1-month follow-ups (B and E), two 6-month follow-ups (C and F), and one re-pre (D) that was a second baseline assessment for Cohort 2 for comparison with a 1-month post of Cohort 1. **B** CONSORT Diagram. Efficacy of WISER intervention: cohort 1 change at 1-month (B–A_1_) vs. cohort 2 control (D–A_2_). Within-cohort change from baseline to 1-month: B–A for cohort 1; E–D for cohort 2; Within-cohort change from baseline to 6-months: C–A for cohort 1; F–D for cohort 2; Effectiveness comparison of cohort 2 change versus cohort 1 at 1-months: (B–A) vs. (E–D); Effectiveness comparison of cohort 2 change versus cohort 1 at 6-month: (C–A) vs. (F–D).

We conducted a pragmatic, cluster randomized controlled trial (RCT) in eight academic levels 4 NICUs randomized to two cohorts of four NICUs each. Each cohort included a mix of NICUs that were either within a free-standing children’s hospital or part of an adult hospital. We selected a clustered design to mitigate the risk of contamination. Cohort 1 received the intervention immediately, while cohort 2 acted as a waitlist control. Enrollment began in June 2016. Cohort 1 received the intervention from August 2016 to January 2017, then cohort 2 received the intervention from March to April 2017. Participants were informed of their start date and follow-up dates shortly after enrollment. We assessed cohorts at four-time points (Fig. 1A). Each cohort received the intervention; therefore, blinding was not feasible. In addition, given the pragmatic nature of this trial, whereby the second cohort received an abbreviated version of the intervention, blinding was also not feasible as part of the evaluation.

### Participants

Participants were HCWs indicating the NICU as their primary location of work. To be eligible, participants had to be employed for at least 4 weeks prior to the trial and dedicate at least 0.4 full-time equivalents to the NICU. HCWs who did not meet eligibility criteria could choose to participate, but their data were not included in the analyses. NICUs were regionally diverse, located in Massachusetts, North Carolina, Tennessee, Texas, New Mexico, and California.

### Intervention

WISER is comprised of six guided well-being modules based on adult learning principles, combining educational material with practice-based learning [20]. Individual modules have been favorably evaluated as brief, feasible, and practical [16–19]. Each module was sent at 7 p.m. local time, introduced with an 8–10 min evidence-based educational video, with simple and engaging reflective activities lasting from 2 to 7 min. Modules were delivered electronically with a thematic introduction and continued in the following order: (1) gratitude, (2) three good things, (3) awe, (4) random acts of kindness, (5) identifying and using signature strengths, and (6) relationship resilience (for details see eAppendix, Section A). Cohort 1 participants were invited to view modules by mobile or email, each introduced monthly and lasting 10 days. Cohort 2 received the intervention in condensed form over the course of 28 consecutive days. The evaluation was performed via electronic survey administration.

### Measures

#### Primary outcome

The primary outcome of burnout was evaluated using a widely used [16, 18, 21–23] 5-item derivative of the emotional exhaustion scale of the Maslach Burnout Inventory [24], shown to have excellent psychometric properties [16–18, 21, 25], external validity [22, 23, 25], and is responsive to interventions [16–18]. According to a psychometric meta-analysis, of the three sub-scales of burnout (emotional exhaustion, depersonalization, and personal accomplishment), emotional exhaustion consistently produces the largest and most consistent Cronbach alpha estimates [26].

To reduce participant respondent burden, we used a 5-item derivative of the original 9-item scale. This 5-item version is reliable (Cronbach α = 0.92) [16–18, 21, 22, 25], predicts the prevalence of disruptive behaviors as well as symptoms of depression [27] and is associated with HCW work-life balance [27]. HCW emotional exhaustion assessments with this 5-item version are also associated with improvement readiness (the capacity of HCWs to initiate and sustain quality improvement initiatives) [25] and the use of Patient Safety Leadership WalkRounds [21]. Importantly, HCW assessments using this scale are consistently responsive to interventions [16–18, 21]. For this study, we will use the terms burnout to describe the general phenomenon and emotional exhaustion in conjunction with its measurement.

For ease of interpretability, we defined a “percent concerning” measure to highlight the proportion of respondents in each cohort reporting undesirable results. We used the established threshold of 50 or higher [16, 21, 22, 25, 27], which reflects “not disagreeing”, on average, to emotional exhaustion items (see eAppendix Section B for detail).

#### Secondary outcomes

Depressive symptoms were assessed via the Center for Epidemiological Studies Depression Scale-10-item version (CES-D10), a psychometrically sound tool for screening respondents for clinical depression [28]. Depression and emotional exhaustion share some features (e.g., exhaustion and impaired concentration) [27, 29], and emotional exhaustion is a risk factor for depression, but emotional exhaustion is generally viewed as an occupational phenomenon, and depression is a psychological condition. Work-life integration was evaluated using the work-life climate scale, which has been used with HCWs and exhibits good psychometrics [22, 23]. Subjective Happiness was evaluated with the subjective happiness scale (SHS), a validated, psychometrically sound, and internationally used scale of global happiness [30, 31]. For further measurement details, see eAppendix, Section B.

The survey also captured respondent characteristics including gender, race/ethnicity, shift type, job position, and years in the specialty. Job positions included attending physician, fellow (trainee) physician, neonatal nurse practitioner, registered nurse, respiratory care practitioner, and others.

### Randomization

NICUs were randomly assigned to immediate intervention or waitlist control through a random number generator using even and odd numbers for assignment.

### Statistical analyses

For comparability, we rescaled outcome measures to 100-point scales. To test our hypotheses (Fig. 1A), we used a generalized linear mixed-effects modeling framework that included fixed effects for time and cohort, and random effects for worksite and participant [32]. To facilitate interpretation of results, we combined the two cohorts and used percent concerning thresholds. This technique is commonly used in safety culture and well-being research when looking across a set of metrics (some positively and some negatively valenced) such that a “low percent concerning”, or a reduction in percent concerning was easier to interpret [1, 16–18, 21, 25]. For context, we display the combined group study results for emotional exhaustion within a cross-sectional sample of 16,797 respondents (of 23,853 invited, response rate 70.4%), from 818 work units in 31 hospitals in Michigan. We also performed a sensitivity analysis, in which we adjusted for gender, race/ethnicity, shift type, job position, and years in the specialty.

All hypothesis tests were conducted in SAS PROC GLIMMIX and included a Kenward–Roger degree of freedom correction [33]. A *p*-value of <0.05 was considered statistically significant. Statistical analyses were performed in SAS version 9.4. EAppendix, Sections C-D provides additional details.

## Results

Enrollment and participation in the trial are shown in Fig. 1B (CONSORT Diagram). In cohort 1, 182 respondents initiated the intervention by clicking a WISER text or email message. Of these, 100 (55%) and 78 (43%) were still participating at 1- and 6-month follow-up, respectively. Based on qualitative comments from participants that the 6-month intervention period was too lengthy, we shortened the intervention for cohort 2, condensing it into 28 text messages over 28 consecutive days. In cohort 2, initiation of the first module improved, with 299 respondents initiating WISER, of which 233 acted as waitlist control, 176 (59%) completed 1-month and 146 (49%) completed 6-month assessment post-intervention. Table 1 displays the characteristics of the study population by cohort and time point. Cohorts 1 and 2 had similar demographics at baseline. No adverse events were reported.

**Table 1:** Characteristics of the study population.

	Cohort 1						Cohort 2							
	Baseline		1-month post		6-month post		Baseline		Waitlist^a^		1-month post		6-month post	
	*n*	%	*n*	%	*n*	%	*n*	%	*n*	%	*n*	%	*n*	%
Total	182	100.0	100	100.0	78	100.0	208	100.0	233	100.0	176	100.0	146	100.0
*Sex*														
Male	20	11.0	13	13.0	6	7.7	8	3.8	6	2.6	6	3.4	^c^	
Female	162	89.0	87	87.0	72	92.3	200	96.2	172	73.8	149	84.7	129	88.4
*Race/ethnicity*^**b**^														
White	126	69.2	74	74.0	57	73.1	182	87.5	199	85.4	154	87.5	132	90.4
Hispanic	14	7.7	6	6.0	^c^		7	3.4	11	4.7	7	4.0	^c^	
African American	6	3.3	^c^		^c^		9	4.3	11	4.7	7	4.0	^c^	
Asian	34	18.7	14	14.0	14	17.9	9	4.3	8	3.4	6	3.4	^c^	
*Typical Shift*														
Days	111	61.0	57	57.0	47	60.3	114	54.8	95	40.8	91	51.7	75	51.4
Evenings/nights	31	17.0	16	16.0	13	16.7	54	26.0	49	21.0	33	18.8	33	22.6
Variable	40	22.0	27	27.0	18	23.1	40	19.2	34	14.6	31	17.6	25	17.1
*Healthcare worker role*														
Physician^d^	48	26.4	31	31.0	22	28.2	31	14.9	33	14.2	28	15.9	20	13.7
Nurse^e^	97	53.3	49	49.0	42	53.8	140	67.3	162	69.5	113	64.2	102	69.9
APP^f^	9	4.9	^c^		^c^		12	5.8	12	5.2	9	5.1	6	4.1
Others^g^	28	15.4	16	16.0	12	15.4	25	12.0	25	10.7	26	14.8	17	11.6
*Work experience in current position*														
<1 year	25	13.7	14	14.0	8	10.3	28	13.5	12	5.2	19	10.8	11	7.5
1–10 years	91	50.0	46	46.0	35	44.9	114	54.8	106	45.5	83	47.2	80	54.8
≥11 years	66	36.3	40	40.0	35	44.9	66	31.7	60	25.8	53	30.1	42	28.8
*Outcome (% concerning)*^h^														
Emotional exhaustion	60.2		46.5		50.0		59.0		55.2		47.2		49.7	
Depression	39.4		35.5		20.6		34.9		34.2		23.4		25.4	
Work-life integration	40.7		40.0		35.9		37.0		41.6		42.1		40.4	
Happiness	55.3		40.0		33.3		52.2		47.6		32.4		28.1	

^a^Updated baseline for waitlist control.

^b^Across cohorts 5 individuals reported other race/ethnicity.

^c^Categories with ≤5 individuals are not reported in order to protect subject privacy. Data may not add up to 100% due to missing data.

^d^Physician includes attending and fellow physicians.

^e^Nurse includes a registered nurse, nurse manager, and charge nurse.

^f^Advance practice provider (APP) includes physician assistant and nurse practitioner.

^g^Other roles include therapist (e.g., respiratory, physical, occupational, and speech therapist), administrative support (e.g., clerk, secretary, and receptionist), clinical support (e.g., CMA, nurses aid), pharmacist, clinical social worker, manager, dietician/nutritionist, student, and others.

^h^Percent concerning rates were calculated using previously published thresholds.

Implementation of WISER demonstrated both efficacy and enduring effectiveness for burnout (emotional exhaustion) supporting the 4 hypotheses (Table 2).

**Table 2:** Effect of WISER intervention (100-point scale) estimated from generalized linear mixed-effects model.

H1: Efficacy of WISER^a^			Effect of WISER within the cohort					H4: Full and condensed intervention similarly effective^d^		
			Cohort	H2: 1-month^b^		H3: 6-month^c^				
Estimate (95% CI)	*P*-value			Estimate (95% CI)	*P*-value	Estimate (95% CI)	*P*-value	Time	Estimate (95% CI)	*P*-value
*Emotional exhaustion*										
−5.27 (−10.44 −0.10)		0.046	C1	−5.64 (−9.59 −1.68)	0.005	−4.85 (−9.25 −0.45)	0.031	1-month	−1.37 (−6.78 4.04)	0.619
			C2	−4.27 (−7.96 -0.57)	0.024	−1.92 (−5.91 2.07)	0.346	6-month	−2.94 (−8.88 3.00)	0.332
−5.21 (−7.92 −2.51)		<0.001	C1 + C2	−3.23 (−6.22 −0.25)	0.034	1.98 (−1.20 5.15)	0.221	NA	NA	NA
*Depression*										
−1.20 (−5.30 2.89)		0.564	C1	−2.14 (−5.28 1.01)	0.183	−6.06 (−9.52 −2.60)	<0.001	1-month	2.72 (−1.55 7.00)	0.212
			C2	−4.86 (−7.76 −1.96)	0.001	−2.50 (−5.64 0.64)	0.118	6-month	−3.56 (−8.23 1.11)	0.135
−3.72 (−5.75 −1.68)		<0.001	C1 + C2	−3.90 (−6.14 −1.67)	<0.001	−0.19 (−2.55 2.17)	0.875	NA	NA	NA
*Work-life integration*										
2.99 (−0.19 6.17)		0.065	C1	5.18 (2.75 7.60)	<0.001	7.33 (4.64 10.01)	<0.001	1-month	1.09 (−2.24 4.43)	0.520
			C2	4.08 (1.80 6.37)	<0.001	2.92 (0.47 5.37)	0.019	6-month	4.40 (0.77 8.04)	0.018
4.57 (2.97 6.17)		<0.001	C1 + C2	5.07 (3.32 6.82)	<0.001	0.50 (−1.36 2.37)	0.596	NA	NA	NA
*Happiness*										
1.37 (−1.60 4.34)		0.366	C1	0.60 (−1.68 2.87)	0.607	−0.03 (−2.55 2.48)	0.979	1-month	1.14 (−1.98 4.25)	0.474
			C2	−0.54 (−2.67 1.59)	0.619	0.81 (−1.48 3.09)	0.489	6-month	−0.84 (−4.24 2.56)	0.628
−0.20 (−1.74 1.34)		0.794	C1 + C2	0.11 (−1.58 1.80)	0.896	0.32 (−1.49 2.13)	0.731	NA	NA	NA

^a^Hypothesis 1: efficacy of WISER: the intervention will improve NICU healthcare worker burnout (emotional exhaustion; primary outcome), depression, happiness, and work-life integration (secondary outcomes) in cohort 1 compared with waitlist control in cohort 2. (C1: 1-month post-baseline)−(C2: waitlist-baseline).

^b^Hypothesis 2: WISER will be effective at 1 month. C1: 1-month post-baseline; C2: 1-month post-waitlist.

^c^Hypothesis 3: effect of WISER will endure at 6 months. C1: 6-month post-baseline; C2: 6-month post-waitlist.

^d^Hypothesis 4: effect of the condensed cohort 2 intervention will not be less effective than the full intervention in cohort 1. At 1-month: (C1: 1-month post-baseline)−(C2: 1-month post-waitlist); At 6-month: (C1: 6-month post-baseline)−(C2: 6-month post-waitlist).

*The intervention will improve NICU HCW burnout (primary outcome), depression, work-life integration, and happiness (secondary outcomes) in cohort 1 compared with waitlist control in cohort 2 (Hypothesis 1):* represents the RCT component of this study. On a 100-point scale, compared with cohort 2 (waitlist control), the WISER intervention in cohort 1 improved emotional exhaustion (− 5.3, 95% CI −10.4 to −0.1, *p* = 0.046) and resulted in improved work-life integration (+3.0, 95% CI −0.2 to 6.2, *p* = 0.065) that was not statistically significant. Depression and happiness were not significantly affected.

The following hypotheses examine the cohorts in a non-randomized fashion: *WISER will be effective at 1 month (Hypothesis 2):* on a 100-point scale, at 1 month in cohort 1, WISER was associated with reduced emotional exhaustion (−5.6, 95% CI −9.6 to −1.7, *p* = 0.005) and improved work-life integration (+5.2, 95% CI 2.8 to 7.6, *p* < 0.001). At 1 month in cohort 2, WISER was associated with reduced emotional exhaustion (−4.3, 95% CI −8.0 to −0.6, *p* = 0.024), depression (−4.9, 95% CI −7.8 to −2.0, *p* = 0.001), and improved work-life integration (+4.1, 95% CI 1.8 to 6.4, *p* < 0.001). Happiness did not change significantly in either cohort.

*The effect of WISER will endure at 6 months (Hypothesis 3):* outcomes at 6 months showed a similar pattern to 1-month results. In cohort 1, emotional exhaustion (−4.8, 95% CI −9.2 to −0.4, *p* = 0.031), depression (−6.1, 95% CI −9.5 to −2.6, *p* < 0.001), and work-life integration (+7.3, 95% CI 4.6 to 10.0, *p* < 0.001) all improved. In cohort 2, WISER was associated with improved work-life integration (+2.9, 95% CI 0.5 to 5.4, *p* = 0.019). Happiness did not change significantly in either cohort.

*The condensed cohort 2 intervention will not be less effective than the intervention for cohort 1 (Hypothesis 4):* no significant differences in improvement were noted between full and condensed cohorts in any of the outcomes.

### Combined cohort analyses

**Fig. 2:**
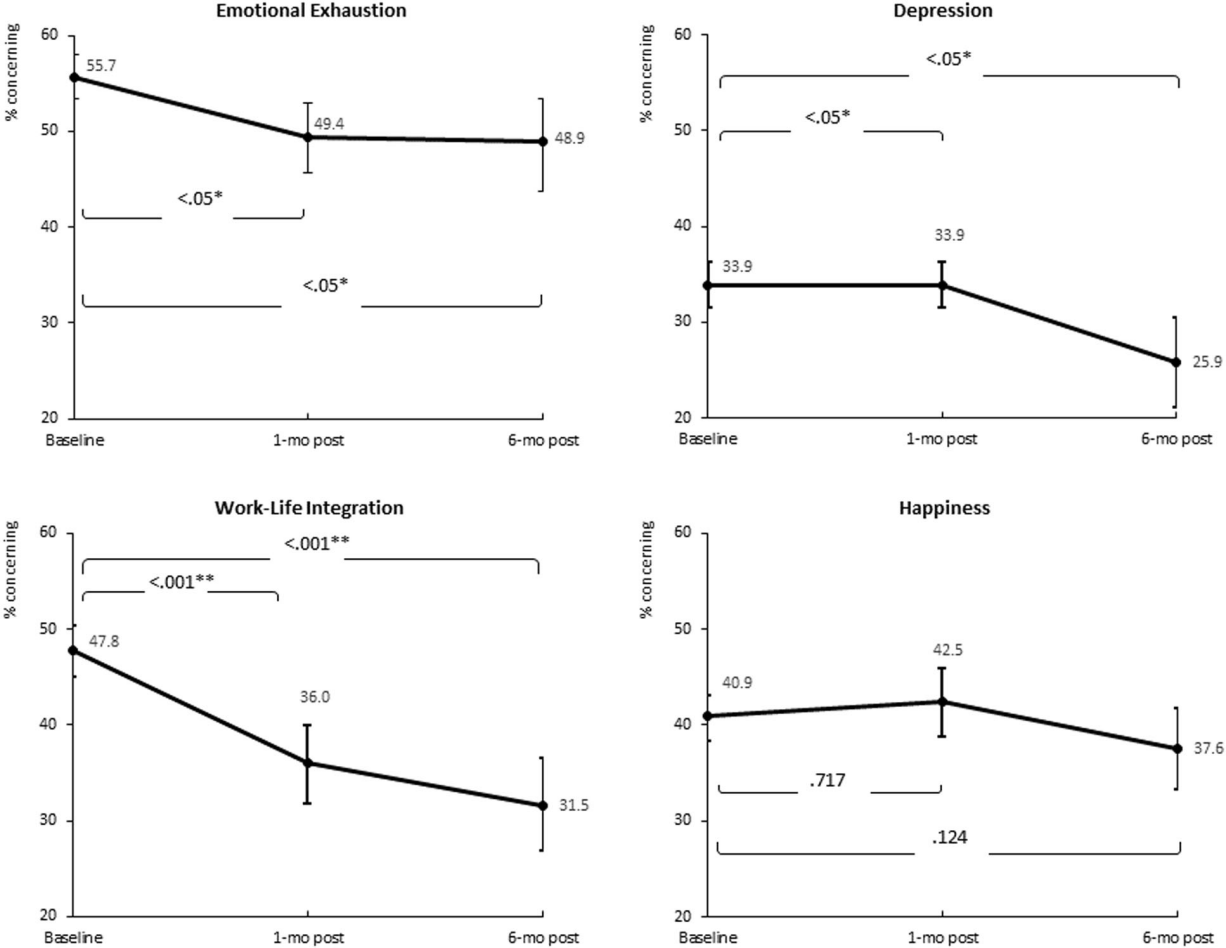
Effect of WISER on the percent concerning scale. Statistical comparisons between combined cohort baseline to 1-month post and 6-month post provided in brackets.

In combined cohorts at 1-month and 6-months on the 100-point scale, WISER was associated with improved emotional exhaustion, depression, and improved work-life integration. Happiness did not change significantly (Table 2). Percent concerning analyses for the combined cohorts similarly showed significant improvement for all metrics except happiness at 1-month and 6-month post-intervention (Table 1 and Fig. 2). Similar results were seen for each cohort on the 100-point scales (eAppendix Figure A).

**Fig. 3:**
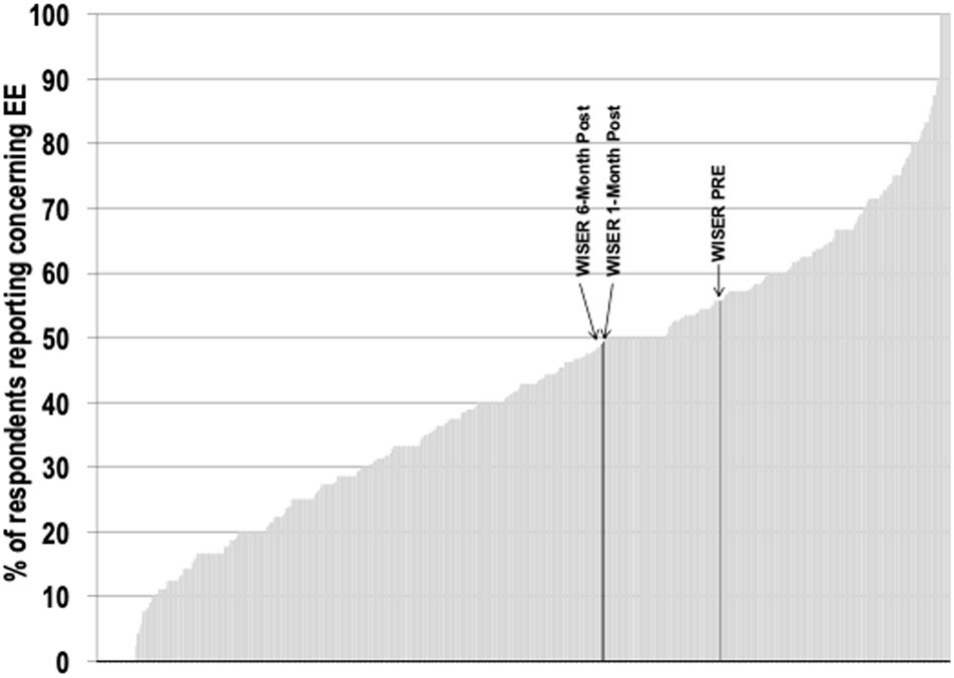
Effect of WISER compared with prior samples. Percent concerning emotional exhaustion in WISER and across 818 work units in 31 hospitals in Michigan. Each bar represents a work unit or WISER study findings. EE - emotional exhaustion. PRE - emotional exhaustion at baseline.

Figure 3 shows the effect of WISER contextualized to a sample of work units in 31 Michigan hospitals. Emotional exhaustion across work units measured varied from 0% to 100% concerning. WISER improved the relative position of our sample of NICUs from 55.7% concerning to 49.4% (1-month post) and 48.9% (6-month post) concerning, equivalent to an improvement from the 73rd percentile to the 59th percentile (lower is better).

In sensitivity analyses, demographic factors (i.e., gender, race/ethnicity, shift type, job position, years in specialty differences) did not differ significantly for initiators compared to non-initiators in all cases, except that nurses in cohort 2 were more likely to initiate WISER compared to other HCWs (eAppendix Table eTA). Additional adjustment for gender, race/ethnicity, shift type, job position, and years in specialty did not change the results (eAppendix Tables eTB/eTC).

## Discussion

In this pragmatic trial, the WISER intervention demonstrated efficacy in reducing burnout (emotional exhaustion) among participating NICU HCWs, compared to a waitlist control at the 1-month primary endpoint. This result was supported by findings in the observational portions of the trial examining the individual cohorts. Six months after completion of the intervention, participants continued to exhibit lower emotional exhaustion. In addition, participation in WISER was associated with improvements in work-life integration and depression both at 1-month and 6-months. Our findings suggest that personal well-being interventions based on positive psychology research may help stem and reverse the rising tide in HCW burnout. This may be especially salient during the current SARS-CoV-2 pandemic, which is overwhelming the well-being landscape of HCWs and will require innovative interventions that can be delivered at scale and on-demand to HCWs that are suffering [34]. Effect sizes for improvement suggest that our results are clinically meaningful, comparing favorably with lengthier and more resource-dependent interventions intended to improve well-being and mental health [16, 35]. Statistical power was strongest in the combined cohort analyses, wherein well-being improvements were significant and durable for emotional exhaustion, depression, and work-life integration.

In this pragmatic trial, we condensed the WISER program in the second cohort based on feedback from participants in cohort 1. The result of this change in intervention delivery is that only the comparison of cohort 1 with the waitlist control, i.e., improved emotional exhaustion, provides causal inference, whereas other comparisons should be viewed as observational. The shortened intervention facilitated the completion of the intervention by more participants. However, although statistical testing revealed that both interventions the 1-month to the 6-month intervention were equally effective, the within-cohort analysis showed some attenuation of the effects 6 months after WISER in cohort 2. Although the means for emotional exhaustion, depression, and work-life integration improved, only work-life integration met the criteria for statistical significance. Potential reasons for the attenuation could relate to the shorter duration of the intervention (28 days vs. 6 months), selective attrition by those less burned out, insufficient power, or external confounding. Additional study is needed to determine the optimal design of WISER, including dose, number, and sequencing of modules. Ideally, larger sample sizes would also allow for subgroup analyses by subtypes of respondents, years of experience, etc.

Despite the well-documented descriptions of burnout in healthcare, few interventions have been tested in randomized trials. The WISER intervention packages tools that promote noticing and savoring positive emotions and are feasible and scalable, in contrast to other available interventions, such as those focusing on meditation [36]. People who suffer from burnout experience decreased ability to notice and savor positive emotions in their lives [29]. Rigorous psychological research has consistently shown that experiencing positive emotion is central to building consequential personal resources like well-being [36], as well as helping to find meaning after adversity [37], and accelerating recovery after emotional upheavals [38]. Experiencing positive emotions has both psychological and physiological benefits, undoing cardiovascular sequelae of emotional upheavals [39].

During the waitlist period, work-life integration improved in the control group. Pilot testing showed similar trends towards improvement in work-life integration when people completed the scale multiple times. It is possible that increasing personal awareness of, e.g., how often one gets less than five hours of sleep, skips meals, and gets home late is itself a subtle intervention.

WISER did not significantly change reported happiness among participants. This finding contrasts a prior cohort study [16] and highlights the need for further research to identify a robust set of well-being metrics for HCWs.

This study should be viewed in light of its design. Well-being interventions, in particular, have much higher attrition and non-initiation than other kinds of RCTs (e.g., drug trials [40]). We similarly experienced this complication which introduced selection bias. We attempted to maximize the pool of potential participants by visiting each NICU to introduce and discuss the study in seminars and on dayshift and nightshift walk rounds. Although almost half of all potentially eligible HCWs expressed interest in WISER, the number who initiated the intervention was considerably smaller; in this study 481 HCWs (out of 1087, 44.3%) initiated WISER. This challenge of low initiation rates among busy HCWs was exemplified in a recent RCT of professional coaching [35] for physicians that showed similar efficacy to our study, although only 88 of 764 eligible physicians chose to participate. The present study compares favorably to this and other interventions, including dieting, smoking cessation, and other web-based well-being interventions [41, 42], which tend to have high rates of non-initiation (~80%) even when financial incentives are provided [43]. Burnout itself may contribute to a lack of initiation energy and may explain the lack of effectiveness found for workplace well-being programs [44]. Despite these limitations, our sensitivity analyses demonstrate that the initiators were not measurably different from those who initially expressed interest in the interventions but did not initiate.

Both study cohorts experienced significant attrition, which may also introduce selection bias. Such attrition is well-described among other behavioral and well-being interventions, which commonly report high discontinuation (33–50%) [45], and significant loss to follow-up (40–48%) [40, 43, 45–48]. It is unknown if participants who were lost to follow-up experienced similar improvements to those who completed the study, suggesting that our results should be interpreted with caution and need to be reproduced in other samples.

Although our study sites were geographically diverse, the participants were mostly white females, reflecting the workforce in many large academic center NICUs. It is uncertain whether our findings are generalizable to other NICUs with more diverse workforces. Future larger samples would ideally allow researchers to tailor WISER modules to specific groups based on their needs, vulnerabilities, and preferences.

## Conclusion

Our study found that WISER showed promise in reducing the emotional exhaustion component of burnout and was associated with significant improvements in other aspects of well-being. Although initiating the intervention among busy HCWs was challenging, participation in a no-cost, low-intensity positive psychology intervention improved burnout, depression, and work-life integration for up to 6 months beyond the intervention. WISER offers healthcare institutions a free, fun, and feasible tool to stem the crisis in HCW burnout and maintain workforce well-being.

### Supplementary information

eAppendix

## Data Availability

The authors will share survey data from the study upon reasonable request to the corresponding author.
