# Supplementary material for: Randomized controlled trial of the “WISER” intervention to reduce healthcare worker burnout
Source: J Perinatol. 2021 Aug 9;41(9):2225–34. doi: 10.1038/s41372-021-01100-y (PMC8440181; doi:10.1038/s41372-021-01100-y)
Supplement: Supplementary file 1 — eAppendix [file 41372_2021_1100_MOESM1_ESM.docx]

**eAppendix**

1. Details of the Intervention ……………………………………………………………......2
2. Details of Measure Scales…………………………………………………………………3
3. Power and Sample Size……………………………………………………………………5

# Statistical Details……………………………………………………………………...…..6

# **eTable**

eTA. Characteristics of the study population by participation status………………..……......9

eTB. Effect of WISER intervention (100-point scale) estimated from generalized linear mixed effects model additionally adjusting for covariates…………………………………..............11

eTC. Effect of WISER intervention (100-point scale) estimated from generalized linear mixed effects model combining two cohorts additionally adjusting for covariates…………12

# **eFigure**

eFA: Effect of WISER on outcomes on the 100-point scale…………………………………13

1. **Details of the Intervention**

WISER intervention modules

*Thematic Introduction* described the prevalence of burnout, evidence of enhancing resilience, and overview of the next six modules. Cohort 1 received 10 reflection activities, and after condensing the intervention for Cohort 2, the number of reflection activities were reduced as detailed below.

*Module 1. Gratitude* provided a structured opportunity to learn about and express gratitude towards others through a guided letter writing exercise.^1,2^ (Cohort 1 X 10 days; Cohort 2 X 2 days)

*Module 2. Three Good Things* introduced a structured opportunity to cultivate positive experiences by reflecting for several minutes each evening on those that occurred that day.^1^ Participants were invited to share their nightly Three Good Things in an electronic log viewable to other participants.^3^ (Cohort 1 X 10 days; Cohort 2 X 10 days)

*Module 3. Awe* provided an opportunity to learn about and experience the benefits of awe and wonder through a series of visually and conceptually stunning images followed by an exercise to look for and document one of their own experiences of awe.^4^ (Cohort 1 X 10 days; Cohort 2 X 2 days)

*Module 4. Random Acts of Kindness* provided an opportunity to learn, practice and reflect on the power of providing unsolicited kindness to others through documenting in a log acts of kindness that were witnessed, committed, and recieved.^5^ (Cohort 1 X 10 days; Cohort 2 X 2 days)

*Module 5. Identifying and Using Signature Strengths* module used a strengths-finder tool to examine what participants do well, and sent daily prompts to elicit how participants will deliberately use their strengths in new and more frequent ways to address their challenges.^1^ (Cohort 1 X 10 days; Cohort 2 X 4 days)

*Module 6. Relationship Resilience* promoted understanding of beneficial relationship patterns, including the emphasis on positive interactions and experiences; participants kept a log of recent conversations where they noted what made it an uplifting interaction.^6^ (Cohort 1 X 10 days; Cohort 2 X 8 days)

The gratitude, awe and random acts of kindness modules were changed from 10 brief reflections (1 to 2 minutes) to 2 longer reflections (5-7 minutes). Signature Strengths was condensed from 10 reflections (2 minutes) to 4 reflections (4-5 minutes). Relationship Resilience was slightly condensed from 10 reflections (1 to 2 minutes) to 8 reflections (1 to 2 minutes). Three Good Things was not condensed for Cohort 2 (10 reflections of 1-2 minutes).

WISER administration

Each module contained a <10-minute long educational video. Participants then received a nightly text or email (based on their preference) that involved engaging approximately 2-8 minutes with an evidence-based resilience program component. Participants were sent access to WISER content at 7pm in their time zone, but could engage with the content at a time they selected. Site champions promoted the effort, provided reminders to the staff, and locally reinforced learning from the videos. To promote participation, participants were able to receive continuing education credits and/or maintenance of certification part 4 credits.

A physician and nurse champion at each site received a daylong face-to-face training in the use of WISER tools and videos. The intervention began for all NICUs with baseline surveys that were completed during an in-person “kick-off” day in which the investigators provided study background and invited participation.

1. **Details of Measure Scales**

*Burnout (primary outcome):* The primary outcome of burnout was evaluated using a widely used^3,7-10^ 5-item derivative of the emotional exhaustion scale of the Maslach Burnout Inventory,^11^ shown to have excellent psychometric properties^3,7,10,12,13^, external validity^8,9,12^, and is responsive to interventions.^3,10,13^ Emotional exhaustion, unlike its sibling domains of depersonalization and low personal accomplishment (from the Maslach Burnout Inventory), heralds unique attributes. First, emotional exhaustion alone has shown adequate reliability for individual-level measurement.^14^ Second, it has been used to discriminate between burned out and non-burned out outpatients suffering from work-related neurasthenia (according to ICD-10 criteria and DSM-IV).^15,16^ Third, according to a psychometric meta-analysis, emotional exhaustion consistently produces the largest and most consistent Cronbach alpha estimates.^17^

For ease of interpretability, we defined a “percent concerning” measure to highlight the proportion of respondents in each cohort reporting undesirable results. We used the established threshold of 50 or higher,^3,7,8,12,18^ which reflects “not disagreeing,” on average, to burnout items. This threshold identifies individuals whose scores represent concerning levels of burnout but is not considered clinically diagnostic.

The question prompts were as follows: (1) I feel fatigued when I get up in the morning and have to face another day on the job, (2) I feel burned out from my work, (3) I feel frustrated by my job, and (4) I feel I am working too hard on my job, (5) Events at work affect my life in an emotionally unhealthy way. The questions were adapted to the survey format of SCORE,^3,7,9,19,20^ which changed its response scale and scoring to range from 1 (disagree strongly) to 5 (agree strongly).

A burnout score for each respondent was computed by taking the mean of the five items (1 to 5 points for each response) and transforming it to a 100-point scale by subtracting 1 from the mean and multiplying by 25. Each individual thus could receive a minimum burnout score of 0 if all responses were “strongly disagree,” a maximum score of 100 if all responses were “strongly agree,” or intermediate scores reflecting any combination of responses.

*Depressive Symptoms:* The Center for Epidemiological Studies Depression Scale-10-item version (CES-D10) is a psychometrically sound tool for screening respondents for clinical depression.^21^ All items are prefaced with, “during the past week, how often did this occur and include items such as “I could not ‘get going’” and “my sleep was restless”. Responses are answered on a four-point scale (0=rarely or none; 3=all of the time). Each participant’s responses are summed together to achieve a 0-30 point scale, and multiplied by 100/30 to transform to the same 100-point scale as burnout.

*Work-life Integration*: Work-life integration items are from the work-life climate scale, which has been shown to have good psychometrics when used on healthcare workers.^9^ Work-life integration items elicit behavioral work-life infractions by asking: During the past week, how often did this occur? Followed by phrases such as: skipped a meal, arrived home late from work or slept less than five hours in a night. The response scale for the work-life climate items ranged from: Rarely or none of the time (less than 1 day); Some or a little of the time (1-2 days); Occasionally or a moderate amount of time (3-4 days); All of the time (5-7 days); and Not Applicable. Work-life climate scale scores were computed by taking the mean of the seven items (maximum score of 4). We first reversed these scores such that higher scores are representative of higher work-life integration; then transformed to the 100-point scale by multiplying 100/4=25.

*Subjective Happiness:* Lyubomirsky and Lepper’s subjective happiness scale (SHS) is a valid, psychometrically sound, and internationally used scale of global happiness.^22,23^ Example items include “In general I consider myself (1=not a very happy person to 7=a very happy person)” and “Compared to most of my peers I consider myself (1=less happy to 7=more happy)”. All four items of the SHS are answered using a seven-point scale, and each participant’s responses were averaged, with higher scores representative of higher subjective happiness. SHS was mapped to the 100-point scale by multiplying by 100/7.

1. **Power and Sample Size**

Power was calculated for the primary outcome. We considered an improvement in emotional exhaustion of at least 10% to be meaningful based on previous safety culture and work-force well-being studies.^3,20,24,25^ In the context of our study, this translated to a decrease in burnout scores from 50/100 to 45/100, a 5-point decline, or an effect size of 0.25, assuming a standard deviation of 20. In an individually randomized study, we would need 253 participants in each arm of the intervention (or m=64 participants in each of 4 NICUs) to have 80% power to detect this effect size. The clustering of individuals within NICUs induces correlation and inflates the necessary sample size by the design effect 1+(m-1)*ICC (intracluster correlation).^26^ We assumed an ICC of 0.0017 based on previous studies,^27^ leading to a design effect of 1.11, and thus a projected target sample size of 1.11*253 = 280 per arm. As non-initiation and attrition of ~50% are common in behavioral intervention trials, we aimed to enroll a final sample size of n=550 per arm.

Recruitment efforts, including an on-site visit, webinars, and flyers, resulted in 1,087 (44.3%) showing interest in the intervention. However, of these, only 481 initiated WISER, reducing our power to detect an effect of the intervention. Our primary comparison for efficacy of WISER involved 100 WISER participants vs. 233 waitlist controls. Assuming a design effect (variance inflation factor) of 1.11, the effective sample size in this comparison was n=90 WISER participants vs. n=209 controls. In this scenario, the power to detect an effect size of 0.25, or a drop in burnout score from 50/100 in controls to 45/100 in WISER (assuming a standard deviation of 20), would be 50%. These sample sizes would have 80% power to detect an effect size of 0.35, or a drop in burnout score from 50/100 in controls to 43/100 in WISER (assuming a standard deviation of 20). Despite these limitations in power due to lower than anticipated initiation, we were able to demonstrate significant improvements in 3 of the 4 outcome measures (burnout, depression, work-life integration). Given our lack of effect of WISER on happiness, a larger sample, as originally planned, would not have changed our findings.

1. **Statistical Details**

Mixed effects hierarchical models accounting for respondent characteristics nested within NICUs were employed to test intervention effects of each cohort against the control condition and against each other. Specifically, we used the following mixed effects modeling framework for time *k = baseline, waitlist, 1-month post, 6-month post* in participant *j = 1, …, n_i_* in NICU *i* = 1, …, 8:

$$Y_{ijk}= \beta_{0}+ \beta_{1}\left( \mathrm{Cohort}=1, \mathrm{Time}_{k}=1 month post \right)+ {\beta_{2}\left( \mathrm{Cohort}=1, \mathrm{Time}_{k}=6 months post \right)+ \beta}_{3}\left( \mathrm{Cohort}=2, \mathrm{Time}_{k}=baseline \right)+ \beta_{4}\left( \mathrm{Cohort}=2, \mathrm{Time}_{k}=waitlist \right)+ {\beta_{5}\left( \mathrm{Cohort}=2, \mathrm{Time}_{k}=1 month post \right)+ \beta_{6}\left( \mathrm{Cohort}=2, \mathrm{Time}_{k}=6 months post \right)+ \gamma}_{i}+\delta_{ij}+\varepsilon_{ijk}$$

where Y_ijk_ is the outcome (e.g., emotional exhaustion), and $\gamma_{i} \sim N(0, \sigma_{\gamma})$ is a NICU specific random effect independent of the participant level random effect $\delta_{ij}\sim N(0, \sigma_{\delta})$ independent of the error term $\varepsilon_{ijk} \sim N(0, \sigma_{\varepsilon})$ (where ~N is shorthand for “is normally distributed”), such that the implied means by time and cohort for the above model are:

| **Cohort** | baseline | waitlist | 1-month post | 6-month post |
| --- | --- | --- | --- | --- |
| 1 | $\beta_{0}$ | n/a | ${\beta_{0}+\beta}_{1}$ | $\beta_{0}+\beta_{2}$ |
| 2 | $\beta_{0}+\beta_{3}$ | ${\beta_{0}+\beta}_{4}$ | $\beta_{0}+\beta_{5}$ | ${\beta_{0}+\beta}_{6}$ |

All hypotheses were testable using the above model. To test hypothesis 1) of trial efficacy we tested the null hypothesis that β_1_= β_4_ - β_3_; for hypothesis 2) within cohort estimates of WISER effectiveness at 1 month were estimated by the parameters β_1_ for Cohort 1 and (β_5_ - β_4_) for Cohort 2; for hypothesis 3) within cohort estimates of WISER effectiveness at 6 months were estimated by the parameters β_2_ for Cohort 1 and (β_6_ - β_4_) for Cohort 2. Thus hypothesis 4) of similar effectiveness of Cohort 2 versus 1 was tested by the null hypothesis of β_1_≤(β_5_ - β_4_) and β_2_≤(β_6_ - β_4_) at 1 month and 6 months, respectively. T-tests with Kenward-Roger degrees of freedom correction in SAS PROC GLIMMIX were used to test all hypotheses.

|  |
| --- |

# **eTable A.** Characteristics of the study population by participation status

|  | **Initiators^*^** | | | | | | | | |  | **Non-Initiators** | | | |  |
| --- | --- | --- | --- | --- | --- | --- | --- | --- | --- | --- | --- | --- | --- | --- | --- |
|  | Baseline | | Waitlist^**^ | | 1-mo post | | 6-mo post | | |  | Baseline | | Waitlist^*^ | |  |
|  | n | % | n | % | n | % | n | % |  | | n | % | n | % |  |
| **Total** | 390 | 100.0 | 233 | 100.0 | 276 | 100.0 | 224 | 100.0 |  | | 109 | 100.0 | 237 | 100.0 |  |
|  |  |  |  |  |  |  |  |  |  | |  |  |  |  |  |
| **Sex** |  |  |  |  |  |  |  |  |  | |  |  |  |  |  |
| Male | 28 | 7.2 | 6 | 2.6 | 19 | 6.9 | 10 | 4.5 |  | | 13 | 11.9 | 13 | 5.5 |  |
| Female | 362 | 92.8 | 172 | 73.8 | 236 | 85.5 | 201 | 89.7 |  | | 96 | 88.1 | 111 | 46.8 |  |
| **Race/ethnicity ^***^** |  |  |  |  |  |  |  |  |  | |  |  |  |  |  |
| White | 308 | 79.0 | 199 | 85.4 | 228 | 82.6 | 189 | 84.4 |  | | 95 | 87.2 | 190 | 80.2 |  |
| Hispanic | 21 | 5.4 | 11 | 4.7 | 13 | 4.7 | 10 | 4.5 |  | | ^†^ |  | 15 | 6.3 |  |
| African American | 15 | 3.8 | 11 | 4.7 | 11 | 4.0 | ^†^ |  |  | | ^†^ |  | 10 | 4.2 |  |
| Asian | 43 | 11.0 | 8 | 3.4 | 20 | 7.2 | 19 | 8.5 |  | | ^†^ |  | 12 | 5.1 |  |
| **Typical Shift** |  |  |  |  |  |  |  |  |  | |  |  |  |  |  |
| Days | 225 | 57.7 | 95 | 40.8 | 148 | 53.6 | 122 | 54.5 |  | | 52 | 47.7 | 60 | 25.3 |  |
| Evenings/Nights | 85 | 21.8 | 49 | 21.0 | 49 | 17.8 | 46 | 20.5 |  | | 17 | 15.6 | 19 | 8.0 |  |
| Variable | 80 | 20.5 | 34 | 14.6 | 58 | 21.0 | 43 | 19.2 |  | | 40 | 36.7 | 45 | 19.0 |  |
| **Healthcare Worker Role** |  |  |  |  |  |  |  |  |  | |  |  |  |  |  |
| Physician ^1^ | 79 | 20.3 | 33 | 14.2 | 59 | 21.4 | 42 | 18.8 |  | | 18 | 16.5 | 33 | 13.9 |  |
| Nurse ^2^ | 237 | 60.8 | 162 | 69.5 | 162 | 58.7 | 144 | 64.3 |  | | 59 | 54.1 | 131 | 55.3 |  |
| APP ^3^ | 21 | 5.4 | 12 | 5.2 | 13 | 4.7 | 8 | 3.6 |  | | 14 | 12.8 | 32 | 13.5 |  |
| Others ^4^ | 53 | 13.6 | 25 | 10.7 | 42 | 15.2 | 29 | 12.9 |  | | 18 | 16.5 | 38 | 16.0 |  |
| **Work Experience in Current Position** |  |  |  |  |  |  |  |  |  | |  |  |  |  |  |
| < 1 year | 53 | 13.6 | 12 | 5.2 | 33 | 12.0 | 19 | 8.5 |  | | 18 | 16.5 | 20 | 8.4 |  |
| 1-10 years | 205 | 52.6 | 106 | 45.5 | 129 | 46.7 | 115 | 51.3 |  | | 63 | 57.8 | 70 | 29.5 |  |
| ≥ 11 years | 132 | 33.8 | 60 | 25.8 | 93 | 33.7 | 77 | 34.4 |  | | 28 | 25.7 | 34 | 14.3 |  |
| **Outcome (% concerning) ^5^** |  |  |  |  |  |  |  |  |  | |  |  |  |  |  |
| Emotional Exhaustion | 59.6 | | 55.2 | | 46.9 | | 49.8 | | |  | 59.8 | | 69.6 | |  |
| Depression | 37.0 | | 34.2 | | 27.7 | | 23.7 | | |  | 47.6 | | 40.1 | |  |
| Work-Life Integration | 53.6 | | 41.6 | | 41.3 | | 29.9 | | |  | 61.0 | | 45.3 | |  |
| Happiness | 38.7 | | 47.6 | | 35.1 | | 38.8 | | |  | 46.8 | | 53.2 | |  |
| ^1^ Physician includes Attending, Staff, Fellow, and resident Physician. | | | | | | | | | | | | | | | |
| ^1^ Nurse includes Registered Nurse, Nurse Manager, and Charge Nurse. | | | | | | | | | | | | | | | |
| ^3^ Advance Practice Provider (APP) includes Physician Assistant and Nurse Practitioner. | | | | | | | | | | | | | | | |
| ^4^ Other roles include Therapist (e.g., Respiratory, Physical, Occupational, and Speech Therapist), Administrative Support (e.g., Clerk, Secretary, and Receptionist), Clinical Support (e.g., CMA, Nurses Aid), Pharmacist, Clinical Social Worker, Manager, Dietician/Nutritionist, Student, and others. | | | | | | | | | | | | | | | |
| ^5^ Percent concerning rates were calculated using previously published thresholds.  ^*^ Initiators included Cohort 1 (N=182) and Cohort 2 (N=299). | | | | | | | | | | | | | | | |
| ^**^ Updated baseline for waitlist control. | | | | | | | | | | | | | | | |
| ^**^ Across initiators and non-initiators 7 individuals reported other race/ethnicity.  ^†^ Categories with ≤ 5 individuals are not reported in order to protect subject privacy. Data may not add up to 100% due to missing data. | | | | | | | | | | | | | | | |

# **eTable B.** Effect of WISER intervention (100-point scale) estimated from generalized linear mixed effects model additionally adjusting for covariates ^*^

| H1: Efficacy of WISER ^1^ | | | Effectiveness of WISER within cohort | | | | | H4: Full and condensed intervention similarly effective ^4^ | | |
| --- | --- | --- | --- | --- | --- | --- | --- | --- | --- | --- |
|  |  |  | Cohort | H2: 1-mo ^2^ | | H3: 6-mo ^3^ | |  |  |  |
| Estimate (95%CI) | P-value | |  | Estimate (95%CI) | P-value | Estimate (95%CI) | P-value | Time | Estimate (95%CI) | P-value |
| **Emotional Exhaustion** | | | | | | | | | | |
| -5.30 (-10.48, -0.12) | | 0.045 | C1 | -5.69 (-9.65, -1.73) | 0.005 | -5.00 (-9.41, -0.59) | 0.026 | 1-mo | -1.42 (-6.84, 4.00) | 0.607 |
|  |  |  | C2 | -4.27 (-7.97, -0.57) | 0.024 | -1.90 (-5.90. 2.09) | 0.349 | 6-mo | -3.10 (-9.05, 2.85) | 0.307 |
| **Depression** | | | | | | | | | | |
| -1.35 (-5.45, 2.75) | | 0.518 | C1 | -2.23 (-5.38, 0.93) | 0.166 | -6.04 (-9.50, -2.57) | <.001 | 1-mo | 2.67 (-1.62, 6.95) | 0.222 |
|  |  |  | C2 | -4.90 (-7.80, -1.99) | 0.001 | -2.53 (-5.67, 0.62) | 0.115 | 6-mo | -3.51 (-8.19, 1.17) | 0.141 |
| **Work-Life Integration** | | | | | | | | | | |
| 3.05 (-0.12, 6.23) | | 0.060 | C1 | 5.19 (2.76, 7.62) | <.001 | 7.34 (4.65, 10.02) | <.001 | 1-mo | 1.06 (-2.28, 4.39) | 0.533 |
|  |  |  | C2 | 4.13 (1.84, 6.42) | <.001 | 2.92 (0.47, 5.37) | 0.020 | 6-mo | 4.42 (0.78, 8.05) | 0.017 |
| **Happiness** | | | | | | | | | | |
| 1.44 (-1.53, 4.41) | | 0.340 | C1 | 0.65 (-1.62, 2.92) | 0.575 | -0.02 (-2.54, 2.50) | 0.986 | 1-mo | 1.17 (-1.95, 4.28) | 0.463 |
|  |  |  | C2 | -0.52 (-2.65, 1.61) | 0.634 | 0.82 (-1.47, 3.10) | 0.484 | 6-mo | -0.84 (-4.24, 2.56) | 0.628 |
| ^1^ Hypothesis 1: Efficacy of WISER: the intervention improves NICU healthcare worker burnout (emotional exhaustion; primary outcome), depression, happiness, and work-life integration (secondary outcomes) in cohort 1 compared with waitlist control in cohort 2. (C1: 1-month post - baseline) - (C2: waitlist - baseline). | | | | | | | | | | |
| ^2^ Hypothesis 2: WISER will be effective at 1 month. C1: 1-month post - baseline; C2: 1-month post - waitlist. | | | | | | | | | | |
| ^3^ Hypothesis 3: Effect of WISER will endure at 6 months. C1: 6-month post - baseline; C2: 6-month post - waitlist. | | | | | | | | | | |
| ^4^ Hypothesis 4: The effect of the condensed cohort 2 intervention not be less effective than the full intervention in cohort 1. At 1-mo: (C1: 1-month post - baseline) - (C2: 1-month post - waitlist); At 6-mo: (C1: 6-month post - baseline) - (C2: 6-month post - waitlist). | | | | | | | | | | |
| ^*^ Model additionally adjusted for gender, position, race/ethnicity, shift type, and working experience compared to the main model. | | | | | | | | | | |

# **eTable C.** Effect of WISER intervention (100-point scale) estimated from generalized linear mixed effects model combining two cohorts additionally adjusting for covariates ^*^

| H1: 1-mo ^1^ | | H2: 6-mo ^2^ | | H3: 6-mo - 1-mo ^3^ | |
| --- | --- | --- | --- | --- | --- |
| Estimate (95%CI) | P-value | Estimate (95%CI) | P-value | Estimate (95%CI) | P-value |
| **Emotional Exhaustion** | | | | | |
| -5.28 (-7.99, -2.57) | <.001 | -3.25 (-6.23, -0.26) | 0.033 | 2.04 (-1.15, 5.22) | 0.210 |
| **Depression** | | | | | |
| -3.80 (-5.84, -1.75) | <.001 | -3.93 (-6.17, -1.69) | <.001 | -0.13 (-2.50, 2.24) | 0.915 |
| **Work-Life Integration** | | | | | |
| 4.63 (3.02, 6.23) | <.001 | 5.05 (3.30, 6.80) | <.001 | 0.42 (-1.45, 2.30) | 0.656 |
| **Happiness** | | | | | |
| -0.15 (-1.70, 1.39) | 0.844 | 0.11 (-1.58, 1.81) | 0.896 | 0.27 (-1.54, 2.08) | 0.772 |
| Three time points were used for both cohorts, 1-mo post, 6-mo post, and Before Intervention. Before Intervention is defined as: baseline for C1; C2: waitlist for C2. | | | | | |
| ^1^ Hypothesis 1: Effect of WISER at 1 month. 1-month post - before intervention. | | | | | |
| ^2^ Hypothesis 2: Effect of WISER will endure at 6-months. | | | | | |
| ^3^ Hypothesis 3: Sustainability from 1-mo post to 6-month post. | | | | | |
| ^*^ Model additionally adjusted for gender, position, race/ethnicity, shift type, and working experience compared to the main model. | | | | | |

# **eFigure A.** Effect of WISER on outcomes on the 100-point scale


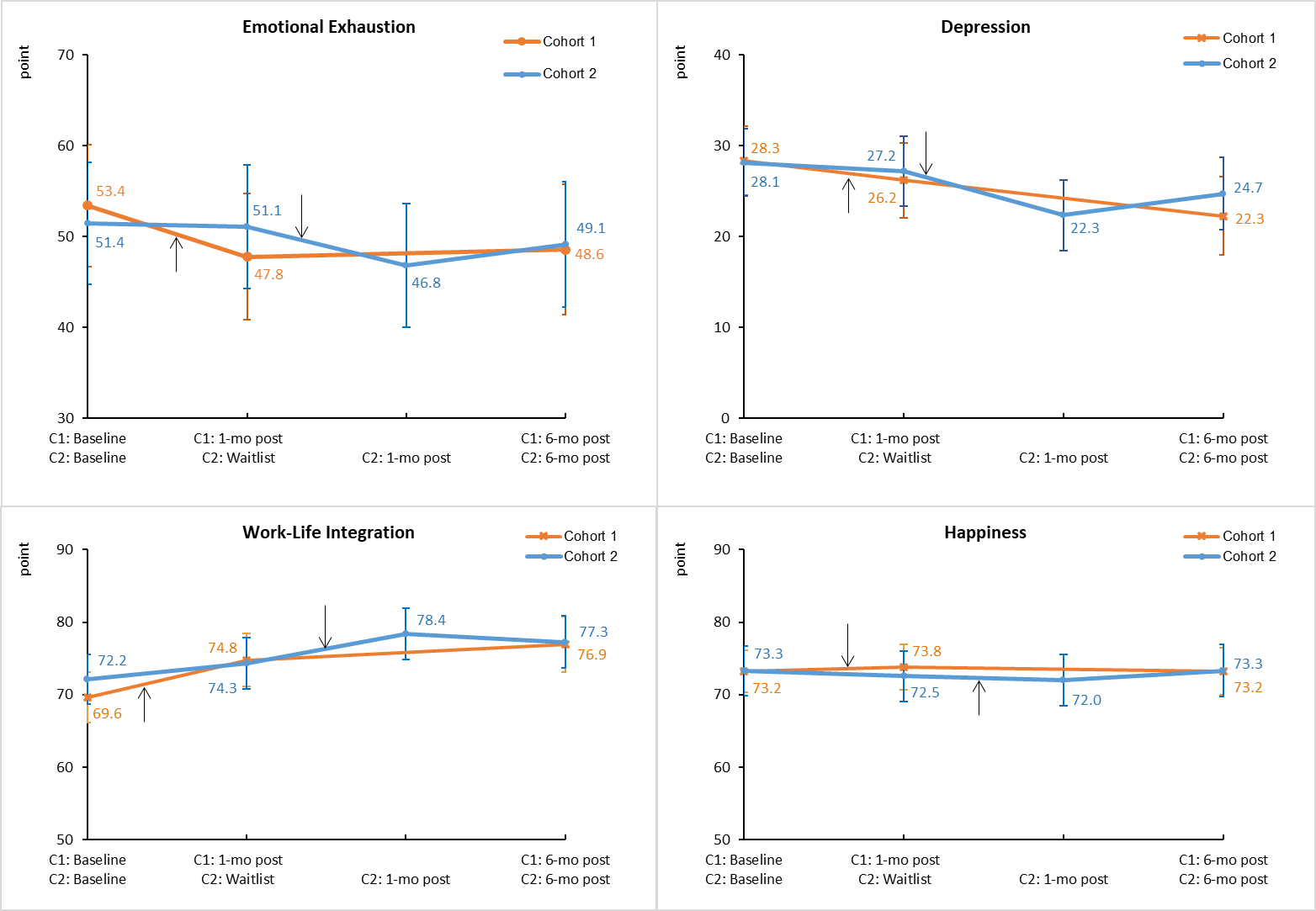


Baseline (both cohorts); Waitlist: Control waitlist before intervention (cohort 2 only); 1-mo post: 1-month post-intervention (both cohorts); 6-mo post: 6-months post-intervention (both cohorts). An arrow indicates intervention period for each cohort (Cohort 1: Baseline to 1-month post; Cohort 2: waitlist to 1-month post).

References

1. Seligman ME, Steen TA, Park N, Peterson C. Positive psychology progress: empirical validation of interventions. Am. Psycho 2005;60:410-21.

2. Emmons RA, McCullough ME. Counting blessings versus burdens: Experimental studies of gratitude and subjective well-being in daily life. J. Pers. Soc. Psychol 2003;84:377-89.

3. Sexton JB, Adair KC. Forty-five good things: a prospective pilot study of the Three Good Things well-being intervention in the USA for healthcare worker emotional exhaustion, depression, work-life balance and happiness. BMJ Open 2019;9:e022695.

4. Rudd M, Vohs KD, Aaker J. Awe expands people's perception of time, alters decision making, and enhances well-being. Psychol Sci 2012;23:1130-6.

5. Pressman SD, Kraft TL, Cross MP. It’s good to do good and receive good: The impact of a ‘pay it forward’ style kindness intervention on giver and receiver well-being. J Posit Psychol 2015;10:293-302.

6. Kok BE, Coffey KA, Cohn MA, et al. How positive emotions build physical health: perceived positive social connections account for the upward spiral between positive emotions and vagal tone. Psychol Sci 2013;24:1123-32.

7. Sexton JB, Adair KC, Leonard MW, et al. Providing feedback following Leadership WalkRounds is associated with better patient safety culture, higher employee engagement and lower burnout. BMJ Qual Saf 2018;27:261-70.

8. Schwartz SP, Adair KC, Bae J, et al. Work-life balance behaviours cluster in work settings and relate to burnout and safety culture: a cross-sectional survey analysis. BMJ Qual Saf 2019;28:142-50.

9. Sexton JB, Schwartz SP, Chadwick WA, et al. The associations between work-life balance behaviours, teamwork climate and safety climate: cross-sectional survey introducing the work-life climate scale, psychometric properties, benchmarking data and future directions. BMJ Qual Saf 2017;26:632-40.

10. Adair KC R-HL, Masoud S, Mosca PJ, Sexton, BJ. Gratitude at Work: A Prospective Cohort Study of a Web-based, Single-exposure Well-being Intervention for Healthcare Workers. J Med Internet Res 2019.

11. Maslach C, Jackson SE. Maslach Burnout Inventory. Palo Alto, CA: Consulting Psychologists Press, Inc.; 1981.

12. Adair KC, Quow K, Frankel A, et al. The Improvement Readiness scale of the SCORE survey: a metric to assess capacity for quality improvement in healthcare. BMC Health Serv Res 2018;18:975.

13. Adair KC KL, Sexton JB. Three Good Tools: Positively reflecting backwards and forward is associated with robust improvements in well-being across three distinct interventions. J Posit Psychol 2020;15:5, 613-622.

14. Brady KJS, Ni P, Sheldrick RC, et al. Describing the emotional exhaustion, depersonalization, and low personal accomplishment symptoms associated with Maslach Burnout Inventory subscale scores in US physicians: an item response theory analysis. J Patient Rep Outcomes 2020;4:42.

15. Kleijweg JH, Verbraak MJ, Van Dijk MK. The clinical utility of the Maslach Burnout Inventory in a clinical population. Psychol Assess 2013;25:435-41.

16. Schaufeli WB, Bakker AB, Hoogduin K, Schaap C, Kladler A. On the clinical validity of the maslach burnout inventory and the burnout measure. Psychol Health 2001;16:565-82.

17. Wheeler DL, Vassar M, Worley JA, Barnes LLB. A Reliability Generalization Meta-Analysis of Coefficient Alpha for the Maslach Burnout Inventory. Educ Psychol Meas 2011;71:231-44.

18. Rehder KJ, Adair KC, Hadley A, et al. Associations Between a New Disruptive Behaviors Scale and Teamwork, Patient Safety, Work-Life Balance, Burnout, and Depression. Jt Comm J Qual Patient Saf 2019.

19. Profit J, Sharek PJ, Amspoker AB, et al. Burnout in the NICU setting and its relation to safety culture. BMJ Qual Saf 2014;23:806-13.

20. Sexton JB, Sharek PJ, Thomas EJ, et al. Exposure to Leadership WalkRounds in neonatal intensive care units is associated with a better patient safety culture and less caregiver burnout. BMJ Qual Saf 2014;23:814.

21. Andresen EM, Malmgren JA, Carter WB, Patrick DL. Screening for depression in well older adults: evaluation of a short form of the CES-D (Center for Epidemiologic Studies Depression Scale). Am J Prev Med 1994;10:77-84.

22. Lyubomirsky S, Ross L. Changes in attractiveness of elected, rejected, and precluded alternatives: a comparison of happy and unhappy individuals. J Pers Soc Psychol 1999;76:988-1007.

23. Howell RT, Rodzon KS, Kurai M, Sanchez AH. A validation of well-being and happiness surveys for administration via the Internet. Behav Res Methods 2010;42:775-84.

24. Dyrbye LN, Shanafelt TD, Gill PR, Satele DV, West CP. Effect of a professional coaching intervention on the well-being and distress of physicians: A Pilot Randomized Clinical Trial. JAMA Intern Med 2019.

25. Hudson D, Sexton J, Thomas E, Berenholtz S. A safety culture primer for the critical care clinician: The role of culture in patient safety and quality improvement. Contem. Crit Care 2009;7:1-14.

26. Hemming K, Girling AJ, Sitch AJ, Marsh J, Lilford RJ. Sample size calculations for cluster randomised controlled trials with a fixed number of clusters. BMC Med Res Methodol 2011;11:102.

27. Kerr J, Rosenberg D, Millstein RA, et al. Cluster randomized controlled trial of a multilevel physical activity intervention for older adults. Int J Behav Nutr Phys Act 2018;15:32.
